# AMPK activation protects cells from oxidative stress‐induced senescence via autophagic flux restoration and intracellular NAD ^+^ elevation

**DOI:** 10.1111/acel.12446

**Published:** 2016-02-18

**Authors:** Xiaojuan Han, Haoran Tai, Xiaobo Wang, Zhe Wang, Jiao Zhou, Xiawei Wei, Yi Ding, Hui Gong, Chunfen Mo, Jie Zhang, Jianqiong Qin, Yuanji Ma, Ning Huang, Rong Xiang, Hengyi Xiao

**Affiliations:** ^1^ Lab for Aging ResearchState Key Laboratory of Biotherapy and Cancer CenterWest China HospitalSichuan University and Collaborative Innovation Center for BiotherapyChengduChina; ^2^ Center of Infectious DiseasesWest China HospitalSichuan UniversityChengduChina; ^3^ Department of Clinical MedicineMedical School of Nankai UniversityTianjinChina

**Keywords:** AMPK, autophagy, oxidative stress, NAD+, senescence

## Abstract

AMPK activation is beneficial for cellular homeostasis and senescence prevention. However, the molecular events involved in AMPK activation are not well defined. In this study, we addressed the mechanism underlying the protective effect of AMPK on oxidative stress‐induced senescence. The results showed that AMPK was inactivated in senescent cells. However, pharmacological activation of AMPK by metformin and berberine significantly prevented the development of senescence and, accordingly, inhibition of AMPK by Compound C was accelerated. Importantly, AMPK activation prevented hydrogen peroxide‐induced impairment of the autophagic flux in senescent cells, evidenced by the decreased p62 degradation, GFP‐RFP‐LC3 cancellation, and activity of lysosomal hydrolases. We also found that AMPK activation restored the NAD ^+^ levels in the senescent cells via a mechanism involving mostly the salvage pathway for NAD ^+^ synthesis. In addition, the mechanistic relationship of autophagic flux and NAD ^+^ synthesis and the involvement of mTOR and Sirt1 activities were assessed. In summary, our results suggest that AMPK prevents oxidative stress‐induced senescence by improving autophagic flux and NAD ^+^ homeostasis. This study provides a new insight for exploring the mechanisms of aging, autophagy and NAD ^+^ homeostasis, and it is also valuable in the development of innovative strategies to combat aging.

## Introduction

Aging is a physiological phenomenon that occurs in all eukaryote and associated with progressing cellular senescence that featured as the growth arrest, impaired function, and declined metabolism (Toussaint *et al*., 2000; Blagosklonny, 2003). Cellular senescence can occur spontaneously *in vivo* and *in vitro*, also can be induced *in vitro* when cells are exposed to oxidative stress, such as hydrogen peroxide (H_2_O_2_) (Chen & Amos, 1994; Toussaint *et al*., 2000). This type of senescence is commonly referred as to oxidative stress‐induced senescence (SIPS).

Adenosine 5' monophosphate‐activated protein kinase (AMPK) serves as a cellular energy sensor, which is composed of a catalytic α subunit and regulatory β and γ subunits (Xiao *et al*., 2011). The role of AMPK in preventing aging/senescence has been suggested in many studies (Apfeld *et al*., 2003; Stenesen *et al*., 2013; Ido *et al*., 2015). AMPK signaling also activates autophagy. The most commonly described mechanism underlying the effects of AMPK on autophagy is suppression of the mTORC1 pathway (Mihaylova & Shaw, 2011; Salminen & Kaarniranta, 2012). Several pharmacological activators of AMPK, such as metformin and berberine, have been characterized, and their potential for the treatment of metabolic, neurodegenerative and other aging‐related diseases is well recognized (Steinberg & Kemp, 2009; Mo *et al*., 2014).

Dysfunctional autophagy has been observed in aging and age‐related diseases (Levine & Kroemer, 2008; Lipinski *et al*., 2010). Autophagy is a homeostatic cellular recycling mechanism responsible for degrading injured or dysfunctional cellular organelles and proteins in all living cells (Mizushima *et al*., 2010). The dynamic process of autophagy is usually surveyed by determining the autophagic flux (Klionsky *et al*., 2012). Growing evidence has indicated that the rate of autophagosome formation/maturation and the efficiency of autophagosome/lysosome fusion decline with age (Mijaljica *et al*., 2010). The methods used to monitor autophagic flux include evaluations of the degradation of p62 protein and assessment of the activity of autolysosomal hydrolases (Klionsky *et al*., 2012), as well as examining the quenching of GFP‐tagged LC3 protein (Kimura *et al*., 2007).

A decline in the nicotinamide adenine dinucleotide (NAD^+^) in cells is another feature of aged organisms (Yoshino *et al*., 2011; Gomes *et al*., 2013). Supplementation with NAD^+^ precursors was shown to ameliorate or reverse the effects of aging in old worms or mice (Gomes *et al*., 2013; Mouchiroud *et al*., 2013). However, the reasons why the NAD^+^ decreases with age are not fully understood. Interestingly, AMPK activation raises the intracellular NAD^+^ concentrations and activates SIRT1 (Cantó *et al*., 2009), which is mediated via an increase in the protein activity and abundance of NAMPT, a key enzyme in the salvage pathway of NAD^+^ synthesis (Brandauer *et al*., 2013). It is currently unclear whether another pathway of NAD^+^ synthesis, the *de novo* pathway, is related to the aging‐associated NAD^+^ decline or whether AMPK plays a role.

To fill the gaps in knowledge regarding the role of AMPK activation in the protection against aging, the following experiments were conducted in this study: (i) confirming the effects of AMPK activation on senescence in our system, (ii) monitoring the effects of AMPK on autophagic flux, (iii) characterizing the effects of AMPK on NAD^+^ synthesis, and (iv) assessing the relationship between autophagy and NAD^+^ homeostasis. Our results indicate that AMPK activity is critical for protecting cells from SIPS, and this role is closely associated with its effect on autophagic flux restoration and the amendment of NAD^+^ homeostasis.

## Results

### The AMPK pathway was inactivated in cells with H_2_O_2_‐induced senescence

**Figure 1:**
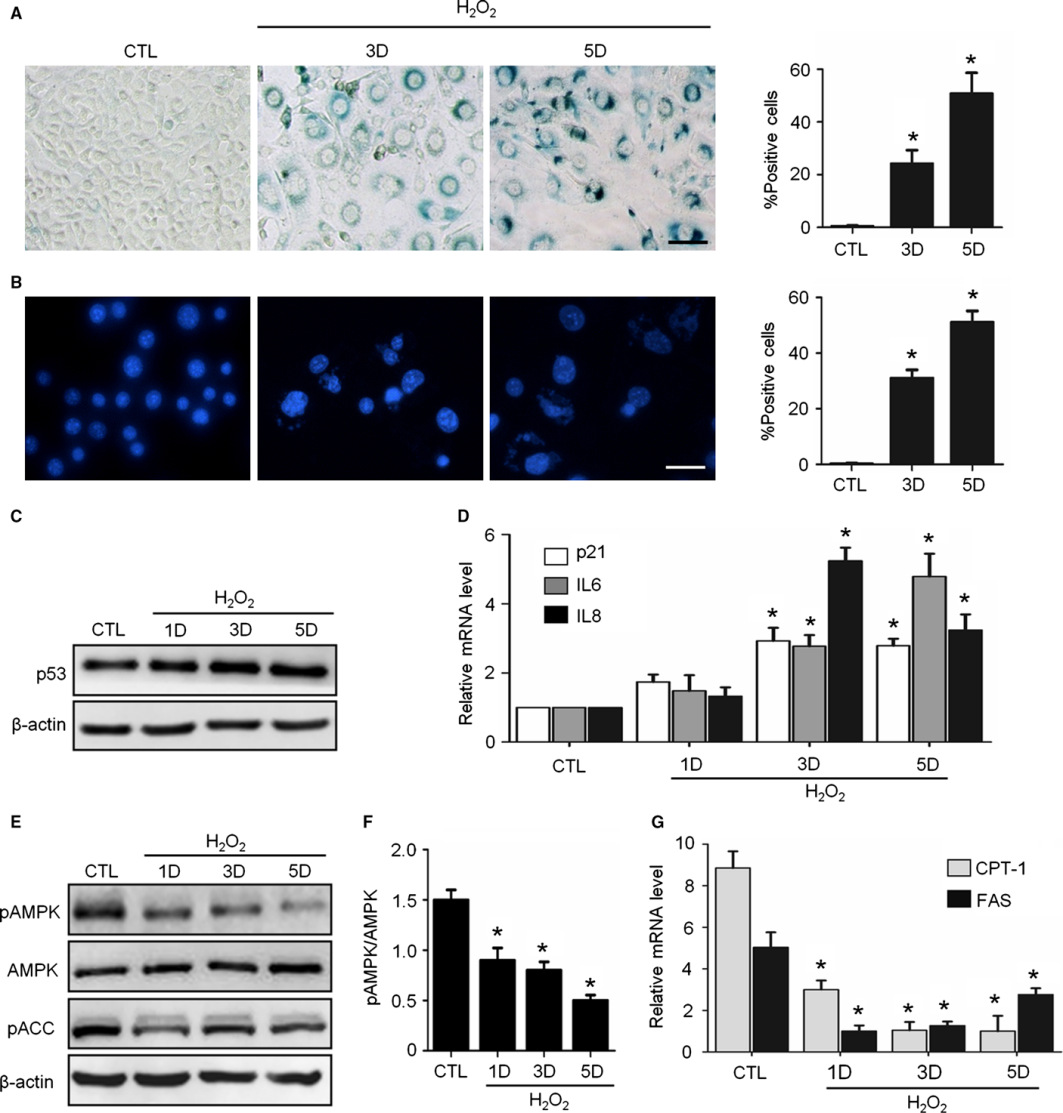
H_2_O_2_ induced senescence and AMPK pathway inhibition in NIH3T3 Cells. Cells were treated with H_2_O_2_ and incubated in complete medium without H_2_O_2_ for 3‐5 days. CTL means untreated cells. (A) Representative images of SA‐β‐Gal staining of the cells (left) and percentages of SA‐β‐Gal‐positive cells in a total of 1000 cells (right). (B) Representative images of SAHFs in cells (left) and percentages of SAHFs‐positive cells in 1000 cells (right). (C) Representative images from immunoblot assays against p53 and β‐actin. (D) Relative fold‐changes in the mRNA levels of the genes encoding *p21, IL6* and *IL8*, as determined by qRT‐PCR. (E) Representative images from immunoblot assays against phosphorylated AMPKα (pAMPK, Thr172), AMPKα1, phosphorylated ACC (pACC, Ser79), and β‐actin. (F) The ratio of pAMPK to total AMPK was quantified by densitometry based on immunoblot images from three independent experiments. (G) Relative fold‐changes in the mRNA levels of two AMPK target genes (*CPT‐1* and *FAS*) were monitored by qRT‐PCR assays. *P < 0.05 compared to the control (CTL). The bar represents 100 μm.

H_2_O_2_ treatment‐induced fibroblast senescence has been widely used as a model of SIPS (Chen & Amos, 1994; Toussaint *et al*., 2000). With a modified procedure, we obtained H_2_O_2_‐induced senescence in NIH3T3 cells with good homogeneity. In brief, suspended cells were treated with H_2_O_2_ for 45 min, and then, they were incubated in complete medium for adhesion culture for five days. At three to five days post‐H_2_O_2_ exposure, the cells became enlarged and flattened morphologically. Strong positive staining for senescence‐associated galactosidase (SA‐β‐Gal) was observed in these cells, with the percentage of SA‐β‐Gal‐positive cells increased by 30 to 50‐fold (Fig. 1A). Another marker of senescence, senescence‐associated heterochromatic foci (SAHFs), was also positive in our H_2_O_2_‐treated cells (Fig. 1B). In addition, the expression levels of four other senescence‐associated genes (p53, *p21*,* IL6,* and *IL8*) were increased in these cells (Fig. 1C,D). The induction of senescence was obtained in the experiments using human MRC‐5 embryonic lung fibroblasts and human umbilical vein endothelial cells (HUVECs) (Fig. S1A,B, Supporting information).

Given that the decline in AMPK activity has been reported to be associated with aging (Salminen & Kaarniranta, 2012), we tested this association in our senescence system. Beginning from the first day after H_2_O_2_ treatment, the levels of phosphorylated AMPKα (Thr172) and phosphorylated ACC (Ser79) markedly decreased, while the protein levels of AMPKα1 remained unchanged (Fig. 1E,F). Similarly, the expression levels of two AMPK target genes, carnitine palmitoyl transferase (*CPT‐1*), and fatty acid synthase (*FAS*), also decreased in the senescent cells (Fig. 1G). These data suggest that the AMPK pathway was downregulated in H_2_O_2_‐induced senescent cells.

### AMPK activation prevented H_2_O_2_‐induced senescence

**Figure 2:**
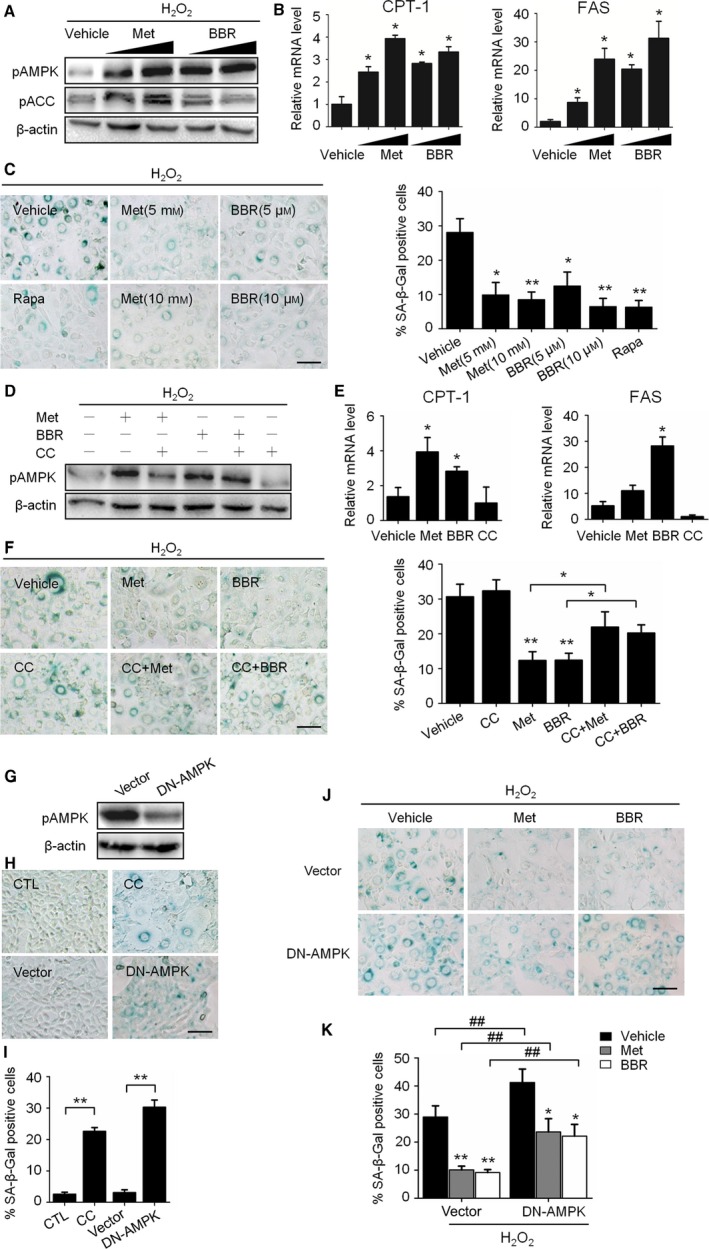
Activation of AMPK prevented H_2_O_2_‐induced senescence. A to D: H_2_O_2_‐treated NIH3T3 cells were incubated in complete medium with metformin (Met, 5 to 10 mM) or berberine (BBR, 5 to 10 μM) for 3 days. (A) Representative images from immunoblot assays against pAMPKα (Thr172), AMPKα1, pACC (Ser79), and β‐actin. (B) Relative fold‐changes in mRNA levels of *CPT‐1* and *FAS* as determined by qRT‐PCR. (C) Representative images of SA‐β‐Gal staining of cells (left), and percentages of SA‐β‐Gal‐positive cells. D‐F: NIH3T3 cells were treated with H_2_O_2_ and incubated with Met (10 mM), BBR (10 μM) and an AMPK inhibitor, Compound C (CC, 10 μM), alone or in combination for 3 days. (D) Representative images from immunoblot assays. (E) Relative mRNA levels of *CPT‐1* and *FAS* as determined by qRT‐PCR. (F) Representative images of SA‐β‐Gal staining of cells (left) and percentages of SA‐β‐Gal‐positive cells (right). G‐I: NIH3T3 cells without H_2_O_2_ treatment were used. (G) The decrease in AMPK activity in DN‐AMPK‐expressing NIH3T3 cells was shown by the decrease in AMPKα phosphorylation. (H) Representative images of SA‐β‐Gal staining of the nontransfected cells with or without CC (upper) and cells transfection with DN‐AMPK (lower). (I) The percentages of SA‐β‐Gal‐positive cells were calculated based on the images represented in H. (J) H_2_O_2_‐treated cells incubated with or without Met (10 mM) or BBR (10 μM) for 3 days, representative images of SA‐β‐Gal staining of cells transfected with empty vector (upper) or DN‐AMPK (lower). (K) The percentages of SA‐β‐Gal‐positive cells were calculated based on the images presented in J. *P <* *0.05 and **P <* *0.01 compared to the vehicle control or indicated sample, ^##^ *P *<* *0.01 compared to the indicated sample. The bar represents 100 μm.

To evaluate the effects of AMPK activation on H_2_O_2_‐induced senescence, two known AMPK activators, metformin (Met) and berberine (BBR), were included in the culture medium after H_2_O_2_ treatment. As shown in Fig. 2A, both Met and BBR significantly enhanced the protein level of pAMPKα and the pACC in senescent cells. In addition, the mRNA levels of *CPT‐1* gene and *FAS* gene increased (Fig. 2B).

Next, we observed that when the H_2_O_2_‐treated cells were incubated with medium containing Met and BBR, there was a dose‐dependent decrease in the percentage of SA‐β‐Gal‐positive cells, which was similar to that caused by rapamycin treatment (Fig. 2C). The effects of AMPK activation on senescence were also evaluated in MRC‐5 cells and HUVECs (Fig. S2A,B). In addition, the preventive effects of AMPK activators were further confirmed using an AMPK inhibitor, Compound C (CC). The efficiency of CC for AMPK inactivation was first confirmed (Fig. 2D,E). As expected, when combined with CC, the effects of Met or BBR on senescence prevention were largely blunted when CC was coexisted, as indicated by the remarkable increase in SA‐β‐Gal‐positive cells (Fig. 2F). These results demonstrate that the activation of AMPK by Met and BBR can prevent H_2_O_2_‐induced senescence, and this prevention could be prevented by CC.

To clarify the role of AMPK in senescence protection_,_ the effects of chronic AMPK inhibition by CC were evaluated in normal cells. As found, many cells incubated with CC for seven days were SA‐β‐Gal‐positive and larger in size compared with the control (Fig. 2H,I), and the cells expressing dominant‐negative AMPKa1 (pDN‐AMPK) also became SA‐β‐Gal positive (Fig. 2G–I). Unsurprisingly, pDN‐AMPK overexpression also increased the SA‐β‐Gal‐positive rate in the H_2_O_2‐_treated cells compared with the cells transfected with the empty vector. Moreover, the antisenescence effects of Met and BBR were weakened in these cells (Fig. 2J,K). These results are consistent with the above findings, confirming the preventive effects of AMPK on senescence.

### AMPK activation restored the H_2_O_2_‐impaired autophagic flux in senescent cells

**Figure 3:**
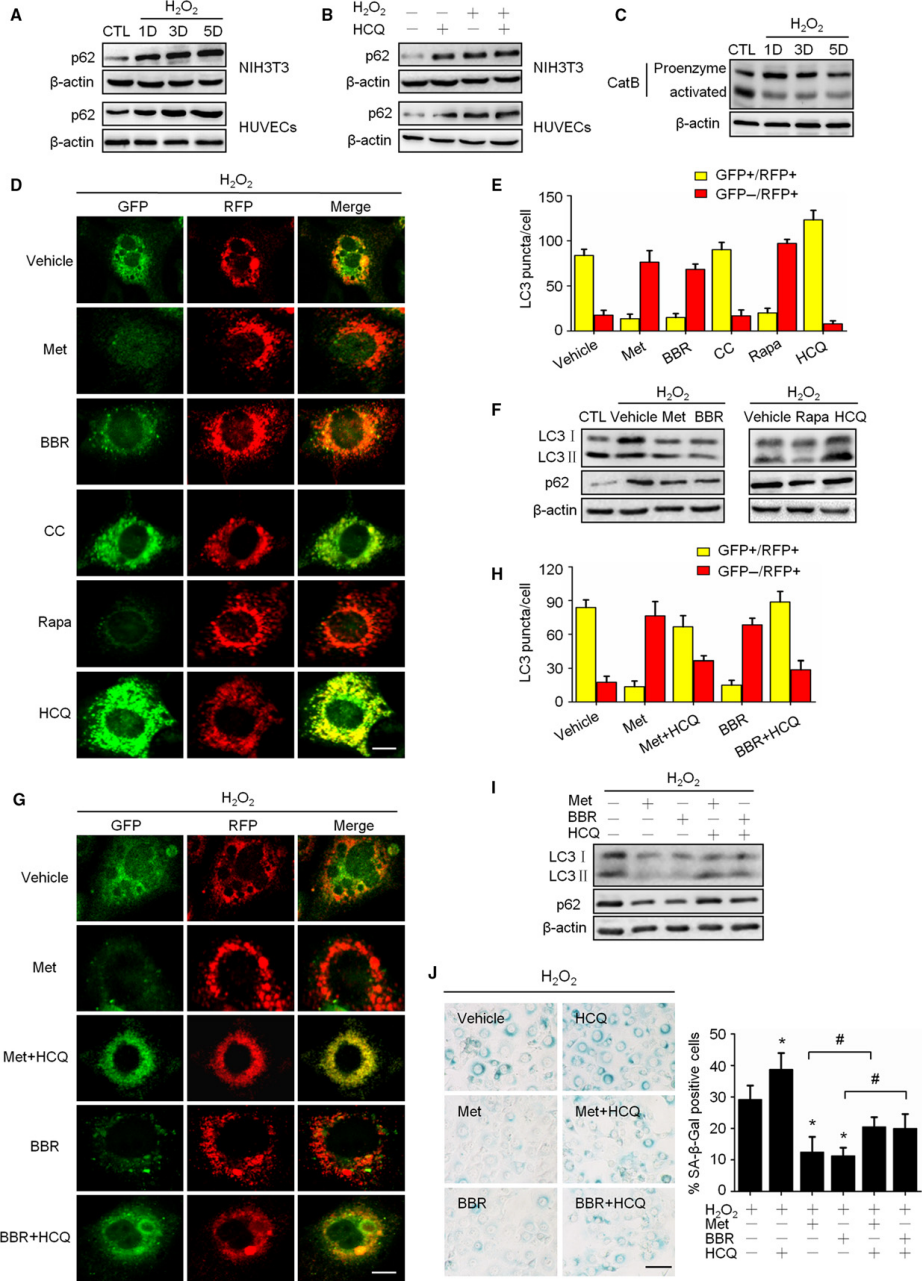
Activation of AMPK improved autophagic flux impaired by H_2_O_2_ treatment. (A) Cells were treated as Fig. 1, representative images from immunoblot assays against p62 and β‐actin. (B) Control and H_2_O_2_‐treated cells were incubated with solvent or hydroxychloroquine (HCQ, 2 μM) for 3 days; representative images from immunoblot assays against p62 and β‐actin. (C) Representative images from immunoblot assays against Cathepsin B protein. D to G: GFP‐RFP‐LC3‐expressing cells were treated with H_2_O_2_ then incubated for 3 days with different reagents including: Met (10 mM), BBR (10 μM), CC (10 μM), Rapa (50 nM), and HCQ (2 μM). (D) Representative confocal fluorescent images of RFP‐GFP‐LC3‐expressing cells, and the right panel shows the merged fluorescence. The bar represents 20 μm. (E) Percentages of cells with puncta like LC3 were figured up based on the images represented in D, dividing into GFP+/RFP+ group (yellow column) and GFP‐/RFP+ group (red column). (F) Representative images from immunoblot assays against LC3 and p62 proteins. (G) GFP‐RFP‐LC3‐expressing cells were incubated with indicated reagents alone or in combination for 3 days; representative confocal fluorescent images are shown as described in D. The bar represents 20 μm. (H) Percentages of cells with punctalike LC3 were figured up and grouped as described in E. (I) Representative images from immunoblot assays against LC3 and p62 proteins. (J) Representative images of SA‐β‐Gal staining of cells (left) and the percentages of SA‐β‐Gal‐positive cells (right). The bar represents 100 μm.*P < 0.05 compared to the vehicle control, ^#^ *P* < 0.05 compared to the indicated sample.

Redressing the autophagic activity is an emerging concept for aging prevention (Rubinsztein *et al*., 2011). With this in mind, we investigated the status of autophagy in senescent cells, paying particular attention to the autophagic flux. The results showed that the p62 protein, dramatically accumulated in H_2_O_2_‐induced senescent cells without an accompanying increase in *p62* mRNA (Figs 3A; S3A). Importantly, different from proliferating cells, the p62 protein did not accumulate when an autolysosomal inhibitor, HCQ, was applied to the senescent cells (Fig. 3B). This implies that almost no autolysosomal degradation capacity remained in the H_2_O_2_‐treated cells, so the inhibitory effects of HCQ in autolysosomes were abrogated. Next, by detecting the protein abundance of Cathepsin B, an important lysosomal protease, we found that the abundance of activated forms of the protein was significantly decreased in H_2_O_2_‐treated cells (Fig. 3C). To examine the status of autophagic flux, a NIH3T3 cell population stably expressing a tandem RFP‐GFP‐LC3 fusion protein was established and employed to visualize and distinguish GFP+RFP+ (yellow) and GFP‐GFP+ (red) LC3 puncta (Klionsky *et al*., 2012). As shown in Fig. S3B, although the formation of LC3 puncta increased in both H_2_O_2_ ‐treated cells and serum‐starved cells, the puncta in H_2_O_2_‐treated cells tended to become GFP+/RFP+ (yellow), while those in starved cells tended to be GFP‐/RFP+ (red). These results reveal that H_2_O_2_‐induced cellular senescence is accompanied by impaired autophagic flux.

Then, the influence of AMPK activity on autophagic flux in senescent cells was investigated via several approaches. First, using RFP‐GFP‐LC3 cells, we found, similar to autophagy activator rapamycin, that Met and BBR weakened the GFP fluorescence in cells. On the contrary, similar to lysosomal inhibitor HCQ, CC enhanced GFP fluorescence and increased yellow LC3 puncta in cells (Fig. 3D,E). Second, with Western blot assay, we found that both Met and BBR alleviated the H_2_O_2_‐induced accumulation of the LC3 and p62 proteins, and this alleviation was consistent with the effect of rapamycin (Fig. 3F). Third, we observed that HCQ markedly blocked the effects of Met and BBR on the promotion of red LC3 puncta (Fig. 3G,H); likewise, HCQ blocked the effects of Met and BBR on the decreases in the LC3 and p62 proteins (Fig. 3I). In fact, blocking autophagic flux by HCQ aggravated H_2_O_2_‐induced senescence and blunted the protective effect of AMPK (Fig. 3J). Fourth, using Atg5‐silenced cells, we found that the influence of autophagic flux blockage the effect of BBR on the protection against senescence (Fig. S4A,B). Finally, we confirmed with HUVECs that BBR can restore autophagic flux and prevent cellular senescence, and this effect can be blunted by HCQ (Fig. S5A,B). Taken together, these findings indicate that AMPK activation by Met and BBR can improve the impaired autophagic flux in H_2_O_2_‐induced senescent cells.

### AMPK restored autophagic flux associated with the amelioration of lysosomal function, mTOR inactivation, but not the nuclear translocation of TFEB

**Figure 4:**
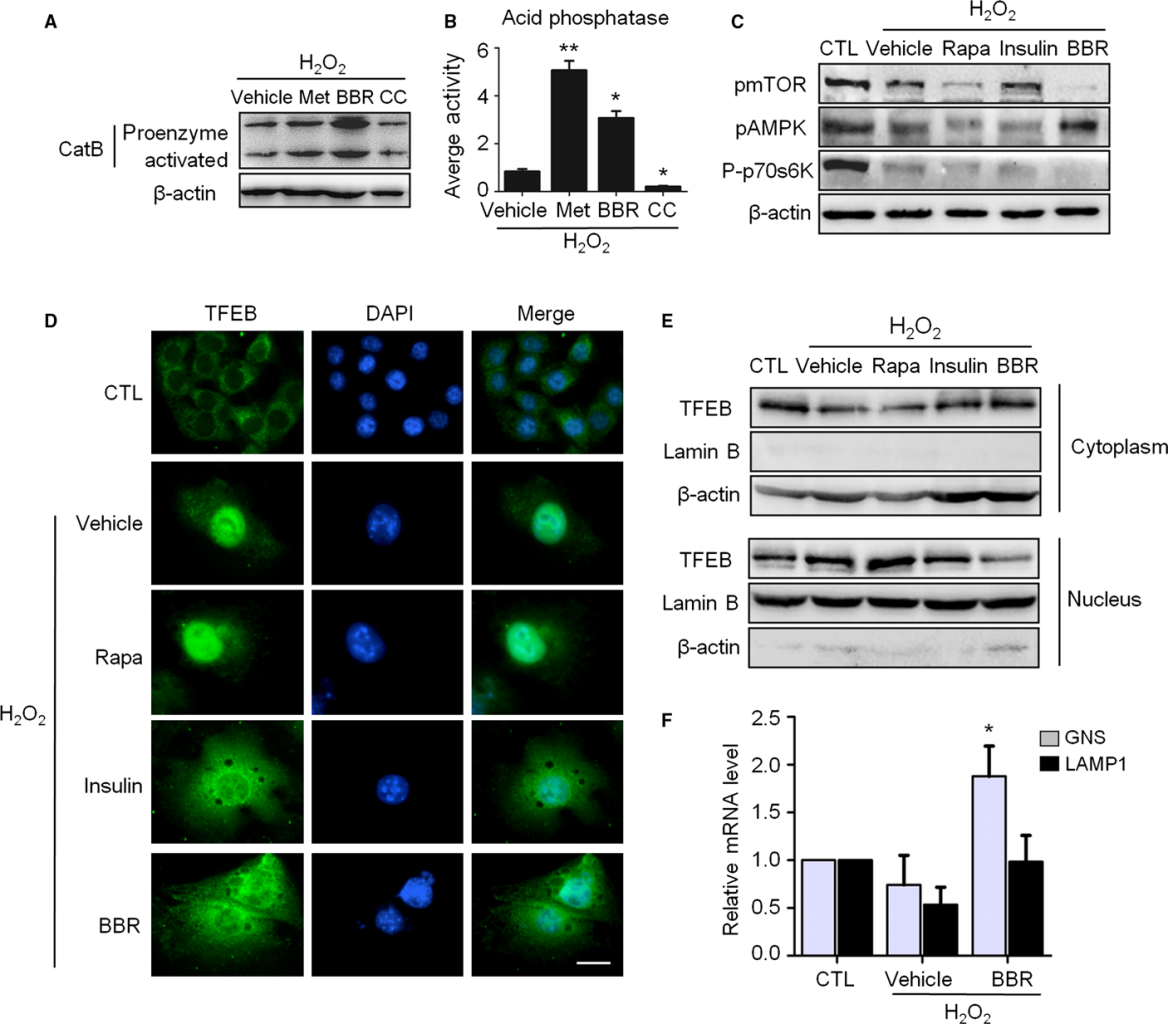
AMPK restored the autophagic flux associated with the amelioration of lysosomal function, mTOR inactivation, but not the nuclear translocation of TFEB. A‐C: H_2_O_2_‐treated NIH3T3 cells were incubated in complete medium with different reagents for 3 days as indicated. (A) Representative images from immunoblot assays against Cathepsin B protein. (B) The acid phosphatase activity in cells. (C) Representative images from immunoblot assays against phosphorylated mTOR (pmTOR, Ser2448), phosphorylated 70S6K (P‐p70S6K, Thr389), pAMPK (Thr172), and β‐actin. (D) Immunofluorescent images of TFEB after treatment with indicated reagents. The bar represents 20 μm. (E) Immunoblot assays against TFEB protein of total cytoplasmic and nuclear subcellular fractions obtained from NIH3T3 cells with the indicated treatment. (F) Relative fold‐changes in mRNA levels of two TFEB target genes (*GNS* and *LAMP‐1*) were monitored by qRT‐PCR assays.*P < 0.05 and **P < 0.01 compared to the vehicle control.

Additional evidence linking AMPK activation to autolysosome restoration was obtained by monitoring the lysosomal functions and the status of the mTOR‐TFEB signaling. The results showed that treatment with Met or BBR increased the abundance of both the proenzyme and activated forms of Cathepsin B (Fig. 4A), as well as the activity of lysosomal acid phosphatase (Fig. 4B). Moreover, we found that BBR treatment significantly suppressed mTOR phosphorylation in senescent cells, similar and even stronger than that induced by rapamycin and insulin as an mTOR activation control (Fig. 4C).

It has reported that mTOR inactivation could result in the release TFEB from the mTORC1 complex following the nuclear translocation of TFEB and elevated transcription of multiple genes related to autophagic activity (Roczniak‐Ferguson *et al*., 2012). However, BBR‐induced mTOR inactivation in H_2_O_2_‐treated cells accompanied without increased nuclear distribution of TFEB, but with the receded (Fig. 4D,E). This situation is different from that observed in rapamycin‐treated cells, where TFEB kept in nucleus (Fig. 4D,E). To know the transcriptional function of nuclear TFEB in H_2_O_2_‐treated cells, we measured the expression of two representative TFEB‐targeted genes, *GNS* and *LAMP1*. We found that the transcription of *GNS* and *LAMP1* genes reduced in H_2_O_2_‐treated cells, but this reduce recovered when BBR applied (Fig. 4F). Above results indicate that the positive effect of AMPK activation on autophagic flux is relevant to its role in combating lysosome dysfunction, and also to mTOR inactivation and TFEB activation as a transcriptional factor. Furthermore, our results reveal that BBR can recede the nuclear accumulation of TFEB induced by H_2_O_2_ treatment.

### AMPK restored NAD^+^ synthesis in cells with H_2_O_2_‐induced senescence

**Figure 5:**
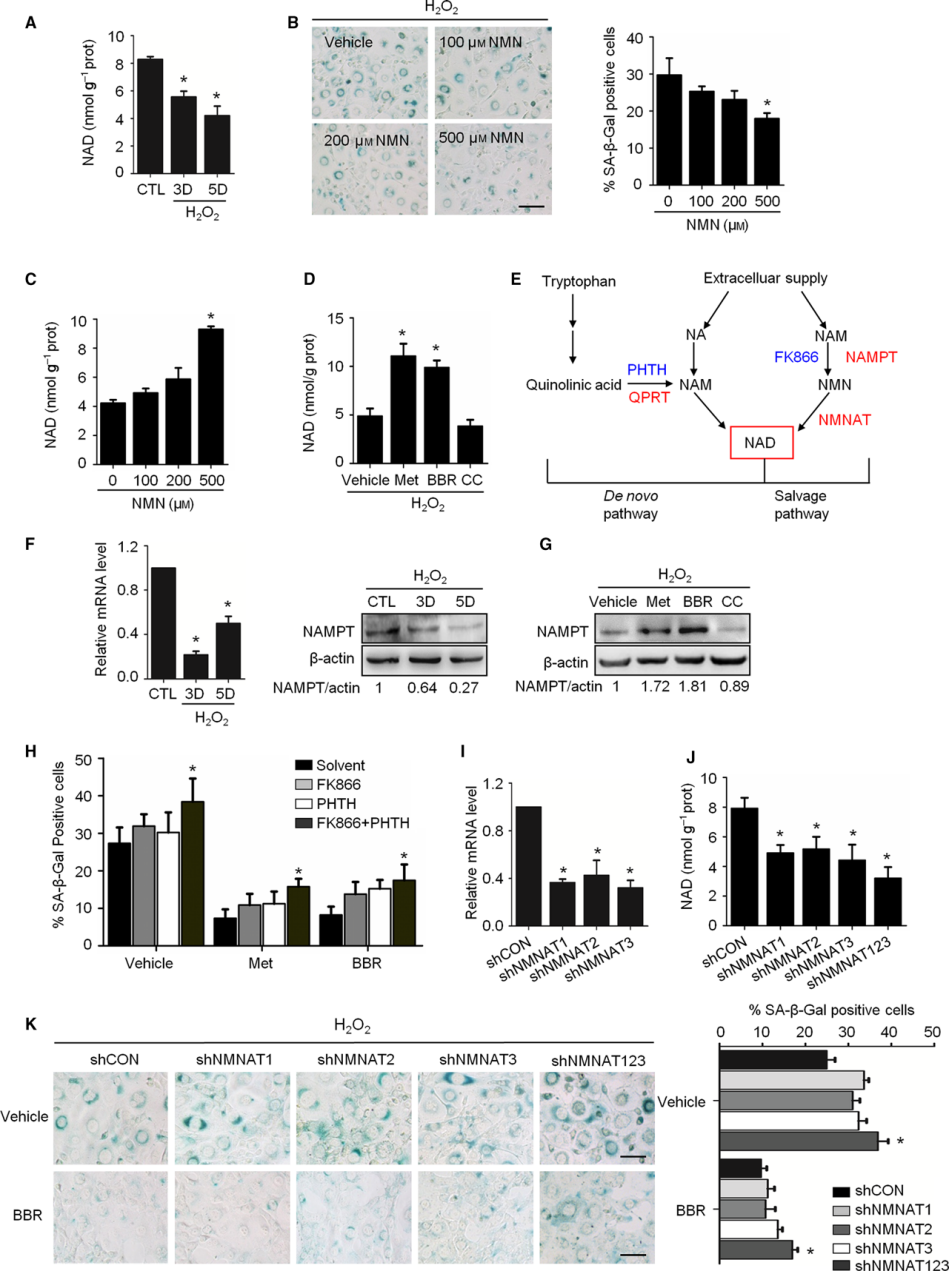
The preventive effects of AMPK on the senescence associated with NAD ^+^ synthesis. (A) Cellular concentrations of NAD ^+^ on day 3 or day 5 after H_2_O_2_ treatment. (B) Representative images of SA‐β‐Gal staining of cells (left) and percentage of SA‐β‐Gal‐positive cells (right). (C) Cellular concentrations of NAD ^+^ in the cells incubated with nicotinamide mononucleotide (NMN) for 3 days after H_2_O_2_ treatment. (D) H_2_O_2_‐treated cells were incubated with Met (10 mM), BBR (10 μM), or CC (10 μM) for 3 days. The concentrations of NAD ^+^ are shown. (E) A schematic diagram of two pathways of NAD ^+^ synthesis. Red ones indicate rate‐limiting enzymes, and blue ones illustrate the enzyme inhibitors we used. (F) Relative fold‐changes in the mRNA levels of *NAMPT*, as monitored by qRT‐PCR (left), and representative images from immunoblot assays against NAMPT are shown (right). The ratio of NAMPT to actin was quantified by densitometry, based on the immunoblot images from three independent experiments. (G) Cells were incubated with Met, BBR, and CC for 3 days. Representative images from immunoblots against NAMPT are shown. The ratio of NAMPT to β‐actin was quantified by densitometry based on the immunoblot images from three independent experiments. (H) H_2_O_2_‐treated NIH3T3 cells were incubated with NAMPT inhibitor (FK866, 5 nM), QPRT inhibitor (PHTH, 1 mM), Met (10 mM), and BBR (10 μM) alone or in combination as indicated for 3 days. The percentages of SA‐β‐Gal‐positive cells are shown. (I) The mRNA levels of *NMNAT1*,*NMNAT2*, and *NMNAT3* genes in the cells infected with corresponding lentivirus‐shRNA or nontargeting shRNA (shCON) as determined by qRT‐PCR. (J) Cellular concentrations of NAD ^+^ in shRNA‐infected cells. (K) Cells infected with shRNAs were treated with H_2_O_2_ and incubated with BBR for 3 days. Representative images of SA‐β‐Gal staining of cells (left) and percentages of SA‐β‐Gal‐positive cells (right). *P < 0.05 compared to the control (A, F) or vehicle (C, D, G, K) or shCON (I, J). The bar represents 100 μm.

Reduced cellular NAD^+^ level is a feature of aging (Gomes *et al*., 2013), and a link between NAD^+^ synthesis and AMPK has been suggested (Brandauer *et al*., 2013). For these reasons, we next examined the relationships among AMPK, NAD^+^ synthesis and senescence. As shown in Fig. 5A, the NAD^+^ level in the senescent cells was significantly decreased, which was accompanied by a decrease in the NAD/NADH ratio (Fig. S6A). Correspondingly, supplementation with nicotinamide mononucleotide (NMN), a precursor of NAD^+^ synthesis (Yoshino *et al*., 2011), decreased the percentage of SA‐β‐Gal‐positive cells and increased cellular NAD^+^ levels following H_2_O_2_ treatment (Fig. 5B,C). Importantly, Met and BBR upregulated the cellular NAD^+^ level, while CC had the opposite effect (Fig. 5D). Similar results were observed using HUVECs (Fig. S7). The ratio of NAD/NADH also increased in the Met‐ and BBR‐treated cells (Fig. S6B).

The involvements of NAD^+^ synthesis assessed next. The known pathways of NAD^+^ synthesis were diagrammed in Fig. 5E. Our results showed that mRNA and protein abundance of nicotinamide phosphoribosyl transferase (NAMPT), a rate‐limiting enzyme of NAD^+^ synthesis in salvage pathway, significantly decreased in senescent cells (Fig. 5F); however, the mRNA level of quinolinic acid phosphoribosyl transferase (*QPRT*), a rate‐limiting enzyme of NAD^+^ synthesis in *de novo* pathway, was increased (Fig. S8A), while no expressional alteration in nicotinamide mononucleotide adenylyltransferase (*NMNAT*) (Fig. S8A). In addition, the situation of NAD^+^ consumption was examined via measuring the activity of a major NAD^+^‐consuming enzyme poly‐ADP‐ribose polymerase (PARP‐1). Resultantly, PARP‐1 activity significantly increased in senescent cells (Fig. S9). These results demonstrate that the NAD^+^ decline found in senescent cells is relevant to both its synthetic decline and consumptive elevation, and for the synthesis, the involvement of salvage pathway seems dominating.

Then, the situation upon AMPK activation was investigated. As shown, the abundance of NAMPT protein and mRNA level of *QPRT* were regulated positively by Met and BBR (Figs 5G, S8B), while no change in the activities of PARP‐1 observed (data not shown). As the effort to know the mechanism about AMPK improved NAD^+^ synthesis, two sets of blocking experiments were performed. The pharmacological approach showed that NAMPT inhibitor FK866 and QPRT inhibitor PHTH‐prompted senescence, but their roles were significantly repressed by Met and BBR (Fig. 5G). The shRNA‐mediated knockdown approach for the *NMNAT* gene showed that each of the three shNMNATs effectively suppressed NMNAT expression (Fig. 5I), and the cellular concentrations of NAD^+^ (Fig. 5J). Moreover, they markedly perturbed the protective role of AMPK activation against senescence (Fig. 5K). These results demonstrate the connection of NAD^+^ synthesis with the AMPK activity in our system and particularly emphasize the involvement of the salvage NAD^+^ synthesis pathway.

Given that NAD^+^ is a coenzyme of Sirt family deacetylases that positively regulates autophagy (Lee *et al*., 2008), and restrain aging (Gomes *et al*., 2013), the activity of Sirt1 was monitored. As the findings, the activity of Sirt1 was increased by AMPK activation in senescent cells (Fig. S10A,B). Then, we noted that EX527, a chemical inhibitor of Sirt1, suppressed the effects of AMPK on senescence and p62 accumulation (Fig. S10C). However, compared with normal cells, the ability of EX527 for blocking the p62 accumulation was only moderate. These results suggest that Sirt1 is involved in H_2_O_2_‐induced senescence as a downstream mediator of AMPK and NAD^+^ biosynthesis.

## Discussion

In this study, we found that autophagic dysfunction and a decline in NAD^+^ are two features of senescent cells induced by oxidative stress, and the activation of AMPK can suppress this type of cellular senescence by restoring both autophagy flux and NAD^+^ synthesis. As AMPK is a key regulator of the metabolic homeostasis in cells, our findings will be informative for more intensive studies of the relationship between AMPK and cellular senescence, which will hopefully contribute to the development of new strategies against organic aging.

The relationship between AMPK and cellular senescence/aging has been suggested (Stenesen *et al*., 2013; Ido *et al*., 2015), and the role of AMPK in aging prevention is generally attributed to its effects on the activation of Sirt1 and FoxO1 (Wang *et al*., 2011; Yun *et al*., 2014), as well as the suppression of mTOR (Salminen & Kaarniranta, 2012). However, in the level of cellular aging, contradictory findings exist (Lee *et al*., 2015). Our results indicate that, at least under oxidative stress, the protective effects of AMPK activation against senescence tend to be predominant. Our observation based on pDN‐AMPKa1 overexpression is also supportive. With regard to the molecular mechanism linking AMPK activation to senescence prevention, several clues have emerged. The best known concept stems from the primary function of AMPK in cells because it generally up‐regulates the generation of ATP synthesis that is important for many cellular processes including autophagy. (Hardie *et al*., 2012). Moreover, the result from Burkewitz *et al*., 2014 is also interesting. It suggests that sustained stimulation of AMPK lead to irreversible senescence, while acute activation of AMPK catabolic pathway permitted a rapid adaptation or resistance to external and internal stresses (Burkewitz *et al*., 2014).

Studies have demonstrated that the antisenescence effects of AMPK are closely involved in the induction of autophagy (Levine & Kroemer, 2008; Salminen & Kaarniranta, 2012), and the physiological aging process is associated with a decline in the efficiency of autophagic degradation, which occurs in autolysosomes and largely limits autophagic flux (Mijaljica *et al*., 2010). Despite the fact that the number of autophagosomes increased in senescent cells, our result strongly supports the notion that autolysosomal degradation and autophagic flux were attenuated in these cells. To explore the status of autophagic flux, we applied several reliable assays (Mizushima *et al*., 2010; Klionsky *et al*., 2012), such as evaluating the Cathepsin B protein level, measuring the acid phosphatase activity, and comparing the influence of a lysosome inhibitor on the p62 accumulation. We also assessed the inhibition of GFP‐LC3 fluorescence using a GFP‐RFP‐LC3‐expressing cell, and obtained consistent data with previous report (Burkewitz K *et al*., 2014), indicating that AMPK activation can improve the autophagic activity in cells with H_2_O_2_‐induced senescence. Particularly, we explored the effects of AMPK activation on the late stage of autophagy, especially on the function of lysosomes, which is different from previous studies that concentrated on the role of AMPK in the early stage of autophagy.

A popular explanation for the association of AMPK with autophagy is its ability to inactivate mTOR pathway (Lerner *et al*., 2013; Burkewitz *et al*., 2014). Recently, transcription factor EB (TFEB) was discovered as a master regulator of lysosomal and autophagic function (Settembre *et al*., 2011), and its nuclear distribution following mTOR inactivation is an accepted mechanistic explanation for the activation of autophagy (Roczniak‐Ferguson *et al*., 2012; Medina *et al*., 2015). According to this mTOR‐TFEB axis theory, mTOR inactivation‐induced TFEB dephosphorylation leads to TFEB translocation to the nucleus, which activates the transcription of specific lysosomal genes (Settembre *et al*., 2011). Unexpectedly, the relationship between mTOR and TFEB in our system does not appear to fit this paradigm. Our results showed that TFEB accumulated abundantly in the nuclei of H_2_O_2_‐induced senescent cells, which have impaired lysosomal function and autophagic flux. Suprisingly, unlike rapamycin, BBR decreased the nuclear localization of TFEB, even it suppressed the mTOR activity. Despite more detailed studies are absolutely needed to elucidate the mechanism(s) underlying these findings, we tend to believe now that the effect of BBR on the lysosomal function in senescent cells is not occur through the classic mTOR‐TFEB axis, and additional regulatory mechanisms affecting the cellular distribution and activity of TFEB may exist.

NAD^+^ levels appear to decline during aging across a broad spectrum of species (Gomes *et al*., 2013; Mouchiroud *et al*., 2013). Our finding that the intracellular NAD^+^ level decreased in SIPS cells is consistent with those *in vivo* studies. As to the mechanism, the elevated consumption of NAD^+^ has been found during aging, particularly relevant to the chronic activation of PARP‐1, which is an NAD^+^‐dependent DNA repair enzyme (Mouchiroud *et al*., 2013). In our model, the activity of PARP‐1 was indeed increased in senescent cells. However, AMPK activators did not suppress this increase (data not shown). On the other hand, we displayed the direct association of the downregulated NAD^+^ synthesis, particularly involving its salvage pathway, with senescence, and also the regulatory effect of AMPK on NAD^+^ synthesis. In the fact, consistent results have been reported by others (Yoshino *et al*., 2011; Brandauer *et al*., 2013;). In addition to the salvage pathway of NAD^+^ synthesis, we also preliminarily addressed the impact on the *de novo* pathway of NAD^+^ synthesis. Based on the increase of *QRPT* mRNA in senescent cells, we assume that *de novo* pathway may compensate the suppressed salvage pathway in H_2_O_2_‐stressed cells for NAD^+^ synthesis. It is worth noting that inhibiting NAD^+^ synthesis, either pharmacologically or genetically, did not obviously decrease the AMPK activation and its role in senescence prevention.

As autophagic dysfunction and a decrease in NAD^+^ are two features of oxidative stress‐induced senescence in cells, it is interesting and important to know their relationship. By reducing NAD^+^ synthesis, either with shNMNATs‐mediated gene silencing or the use of chemical inhibitors of NAD^+^ synthesis, we found that the inhibition of NAD^+^ synthesis in normal cells could obviously suppress the autophagic flux (Fig. S11A,B), suggesting that NAD^+^ homeostasis is required for the maintenance of the autophagic flux. According to the results we obtained and those published by others (Fig. S10D) (Lee *et al*., 2008; Ou *et al*., 2014), we think that a conceivable molecular link between NAD^+^ synthesis and autophagic activation is the Sirt family proteins because NAD^+^ works as a critical coenzyme of Sirt deacetylases, and Sirt has been confirmed to have a role in activating autophagy. Although it is currently unclear why adding NMN cannot restore the autophagic flux in H_2_O_2_‐treated cells (Fig. S11), the damage occurred on the component (s) important for the function of autophagy might be responsive. It should be noted that opposite demonstration has been published previously, saying that the downregulation of cellular NAD^+^ can promote autophagy (Billington *et al*., 2008; Cea *et al*., 2012). We noticed that, however, those previous observations are all based on the use of malignant cells. It is wondering whether the different outcomes observed in our system and in their systems are caused by the quite different cellular situations in normal cells and in malignant cells. For example, the quite high energy requirement of malignant cells may alter the sensitivity of cells to NAD^+^ depletion and consequent ATP production that is necessary for autophagy processing (Khan *et al*., 2007).

In summary, using a H_2_O_2_‐induced senescence model, we were able to provide evidence that the antisenescence effect of AMPK rely on both the activation of autophagy and the restoration of NAD+ synthesis, therefore suggesting that AMPK targets multiple pathways in cells, to collaboratively prevent oxidative stress‐induced senescence. The study is unique due to its emphasis on the autolysosome/lysosome function at the late stage of autophagy and its evaluation for the pathways of NAD^+^ synthesis. Our study also comes up with the interesting links among AMPK activation, autophagy and NAD^+^ homeostasis. These links would be valuable to better understand the senescence and aging, as well as for establishing new antiaging strategies.

## Experimental procedures

### Reagents

Metformin and berberine were purchased from MUSTBIO technology (Chengdu, China); Compound C was from CALBIOCHEM (Darmstadt, Germany). Hydroxychloroquine (HCQ), rapamycin, and phthalic acid (PHTH) were from Sigma‐Aldrich (CA, USA). Nicotinamide mononucleotide (NMN) and FK866 were from Santa Cruz Biotechnologies (TX, USA). EX527 was from selleck (CA, USA). Antibodies against LC3 (12741), phospho‐ACC (Ser79) (3661), phospho‐mTOR (Ser2448) (5536), phospho‐p70S6K (Thr389) (9206), and p53 (2524) were purchased from Cell Signaling technology (MA, USA). Those against phospho‐AMPKα (Thr172) (ab133448), Cathepsin B (ab30443), NAMPT (ab109210), AMPKα1 (ab32047), and SQSTM1/p62 (3340‐1) were from Abcam (MA, USA). Antibodies against β‐actin and Lamin B were from Proteintech (Beijing, China), anti‐TFEB (A303‐673A‐M) was from Bethyl (TX, USA), FITC‐goat anti‐rabbit IgG was from Invitrogen (CA). The reagent used for cDNA plasmids transfection was Lipofectamine 2000 (Invitrogen, CA), and that for lentivirus‐shRNA plasmids was X‐treme GENE HP (Roche, CA, USA).

### Cell culture and H_2_O_2_ treatment

NIH3T3 cells (murine fibroblast line) and MRC‐5 cells (human fibroblast line) were purchased from Shanghai Institutes for Biological Sciences of Chinese Academy of Sciences (Shanghai, China), cultured in complete Dulbecco's modified Eagle's medium (DMEM) supplemented with 10% FBS in a humidified atmosphere with 5% CO_2_ at 37 °C. HUVECs (human umbilical vein endothelial cells) (ATCC) cultured in low glucose (5.6 mM) RIPA1640 supplemented with 10% FBS. For senescence induction, a modified H_2_O_2_ treatment protocol was used. In brief, cells seeded in 100‐mm dishes with 5 × 10^5^ cells per dish density were trypsinized and suspended in phosphate buffer solution (PBS) at 1 × 10^6^ cells mL^−1^ density and exposed to 400 μM (NIH3T3) or 300 μM (MRC‐5) or 700 μM (HUVECs) H_2_O_2_ in an Eppendorf tube at 37 °C for 45 min. During H_2_O_2_ treatment, the tube was turned upside down gently every 5 min. H_2_O_2_ treatment was terminated by a 5‐min centrifugation at 800 rpm and a washing process. Then, the cells were cultured with complete medium. During the adhesion cultivation, the cells accepted different treatments that will be described later in individual figure legends.

### SA‐β‐Gal staining and SAHFs staining

Intracellular senescence‐associated‐β‐galactosidase (SA‐β‐Gal) activity was assayed using an SA‐β‐Gal staining kit (Beyotime, Beijing) according to manufacturer's instructions, and senescent cells were identified as bluish green‐stained cells under a phase‐contrast microscope. The percentage of SA‐β‐Gal‐positive cells in total cells was determined by counting 1000 cells in 7 random fields, for each group. Senescence‐associated heterochromatic foci (SAHFs) was visualized by DAPI staining after cells were fixed *in situ* with 4% paraformaldehyde and washed by PBS. Images with DAPI stained nuclei with blue fluorescence were taken by fluorescence microscope. The percentage of SAHF‐positive cells was determined by counting more than 1000 cells in 7 random fields, for each group. The results were expressed as mean of triplicates ± SD.

### Drug treatments

For AMPK activity modulation, metformin, berberine, and Compound C were applied as described in the legends. For autophagy modulation, rapamycin and hydroxychloroquine (HCQ) were added as described in the legend of Fig. 3. For NAD^+^ precursor supplementation, nicotinamide mononucleotide (NMN) was applied as described in the legend of Fig. 5. For inhibiting NAD^+^ synthesis pathways, FK866 and phthalic acid (PHTH) were applied as described in the legend of Fig. 5.

### mRFP‐GFP‐LC3 expressing cells generation and fluorescent LC3 puncta analysis

Cells were transfected with mRFP‐GFP‐LC3 plasmid (tfLC3 from addgene), and G418 (Life Technology, CA, USA) was added for selecting positive cells. As intracellular distribution of LC3 protein was tagged by the fluorescence of RFP and GFP in these cells, images were collected with fluorescent confocal microscope. Quantification of LC3 puncta was performed using Red and Green Puncta Colocalization Macro with image j program, as described (Mizushima *et al*., 2010), and the average numbers of LC3 puncta per cell were accounted from the data collected from more than 40 cells. Here, GFP+RFP+ puncta are yellow, and GFP‐RFP+ puncta are red. Experiments were repeated three times.

### Intracellular NAD^+^ level measurement

NAD^+^, NADH, and [NAD+]/[NADH] ratio were measured from whole cells extracts using an NAD^+^/NADH quantification kit from AAT Bioquest based on enzymatic cycling reaction, according to manufacturer's instructions (AAT‐15258, CA, USA). The value was normalized according to protein concentrations. Experiments were repeated three times.

### Real‐time PCR analysis

Total RNA was isolated from cultured cells using TRIzol (Takara), and 2 μg of total RNA was used for reverse transcription by QuantiTect Reverse Transcription Kit (Bio‐Rad). Quantitative real‐time polymerase chain reaction (qRT‐PCR) was performed using SYBR Green Supermix kit (Bio‐Rad, CA, USA) on a Bio‐Rad IQ5 system. PCRs were performed in triplicate, and the relative amount of cDNA was calculated by the comparative CT method using the 18S ribosomal RNA sequences as control. The primer sequences used for PCR are shown in Table S1 (Supporting information). Experiments were repeated three times.

### Immunoblotting

Whole cell lysates were collected using ice‐cold lysis buffer (50 mM Tris‐base, 1 mM EDTA, 1 mM EGTA, 150 mM NaCl, 0.1% SDS, 1% TritonX‐100, 1% Sodium deoxycholate, 1 mM PMSF, 1 mM DTT, and 1 mM protease inhibitor) and lysis for 30 min following by centrifugation. Protein concentration was determined by BCA method (Cwbio, China). And 2.5 × SDS loading buffer was added to the lysates following 10 min of boiling. Thirty μg of proteins was loaded on SDS‐PAGE gel and separated by electrophoresis, followed by blotting on a PVDF membrane (Millipore, Germany). The target proteins were probed by corresponding primary antibodies with optimized conditions and then incubated with the secondary antibody. Immunological signals were surveyed via electrochemical luminescence method, using Immobile Western Chemiluminescence HRP substrate kit (Millipore) and Fusion Solo Imaging System (VIBER LOURMAT, FRANCE). The band intensities were quantified by fusion‐capt analysis Software, VILBER LOURMAT, VALLEE, FRANCE. Experiments were repeated three times.

### Statistical analysis

Data were analyzed by by one way ANOVA. Statistical analysis was performed using spss 17.0 software (SPSS, Inc, NY, USA). Error bars represent standard error of the mean (± SEM).

## Funding

This work was supported by National Natural Science Foundation of China (Grant Number 81273224), National 973 Basic Research Program of China (Grant Number 2013CB967204 and Grant Number 2013CB911300).

## Author contributions

Han X carried out most of the experiments, analyzed the data, prepared the figures, and wrote the draft of the manuscript. Tai H, Wang X, Wang Z, Zhou J, Wei X, Ding Y, Gong H, Huang N, Zhang J and Qin J performed some experiments or contributed to data analysis and manuscript preparation. Ma Y and Xiang R contributed to study design. Xiao H conceived and designed the concept of this study, discussed the results with all authors, and worked for the manuscript preparation. The authors declare that they have no conflict of interest.

## Conflict of interest

None declared.

## Supporting information


**Fig. S1** H_2_O_2_ induced senescence in MRC‐5 cells and HUVECs.
**Fig. S2** Activation of AMPK prevented H_2_O_2_‐induced senescence in MRC‐5 cells and HUVECs.
**Fig. S3** H_2_O_2_ induced decreased autophagic flux in NIH3T3 Cells.
**Fig. S4** Atg5 knockdown attenuated the effects of BBR on protection against senescence.
**Fig. S5** Activation of AMPK suppressed the impairment of H_2_O_2_‐induced autophagic flux and decreased the senescence in HUVECs.
**Fig. S6** Activation of AMPK improved the redox status in senescent cells.
**Fig. S7** Activation of AMPK increased the NAD^+^ level in HUVEC Cells.
**Fig. S8** The mRNA level of QPRT increased during senescence.
**Fig. S9** The activity of PARP‐1 increased during senescence.
**Fig. S10** Sirt1 is involved in H_2_O_2_‐induced senescence as a downstream mediator of AMPK and NAD^+^ biosynthesis.
**Fig. S11** NAD^+^ homeostasis is required for maintaining the autophagic flux in normal cells, but not in senescent cells.
**Fig. S12** Unprocessed images of western‐blot.

**Table S1** Primers for real‐time qRT‐PCR.

**Appendix S1** Extended Experimental Procedures.
