# Supplementary material for: AMPK activation protects cells from oxidative stress‐induced senescence via autophagic flux restoration and intracellular NAD + elevation
Source: Aging Cell. 2016 Feb 18;15(3):416–27. doi: 10.1111/acel.12446 (PMC4854918; doi:10.1111/acel.12446)
Supplement: Supplementary file 1 — Fig. S1 H2O2 induced senescence in MRC‐5 cells and HUVECs. Fig. S2 Activation of AMPK prevented H2O2‐induced senescence in MRC‐5 cells and HUVECs. Fig. S3 H2O2 induced decreased autophagic flux in NIH3T3 Cells. Fig. S4 Atg5 knockdown attenuated the effects of BBR on protection against senescence. Fig. S5 Activation of AMPK suppressed the impairment of H2O2‐induced autophagic flux and decreased the senescence in HUVECs. Fig. S6 Activation of AMPK improved the redox status in senescent cells. Fig. S7 Activation of AMPK increased the NAD+ level in HUVEC Cells. Fig. S8 The mRNA level of QPRT increased during senescence. Fig. S9 The activity of PARP‐1 increased during senescence. Fig. S10 Sirt1 is involved in H2O2‐induced senescence as a downstream mediator of AMPK and NAD+ biosynthesis. Fig. S11 NAD+ homeostasis is required for maintaining the autophagic flux in normal cells, but not in senescent cells. Fig. S12 Unprocessed images of western‐blot. [file ACEL-15-416-s001.pdf]

Figure S1

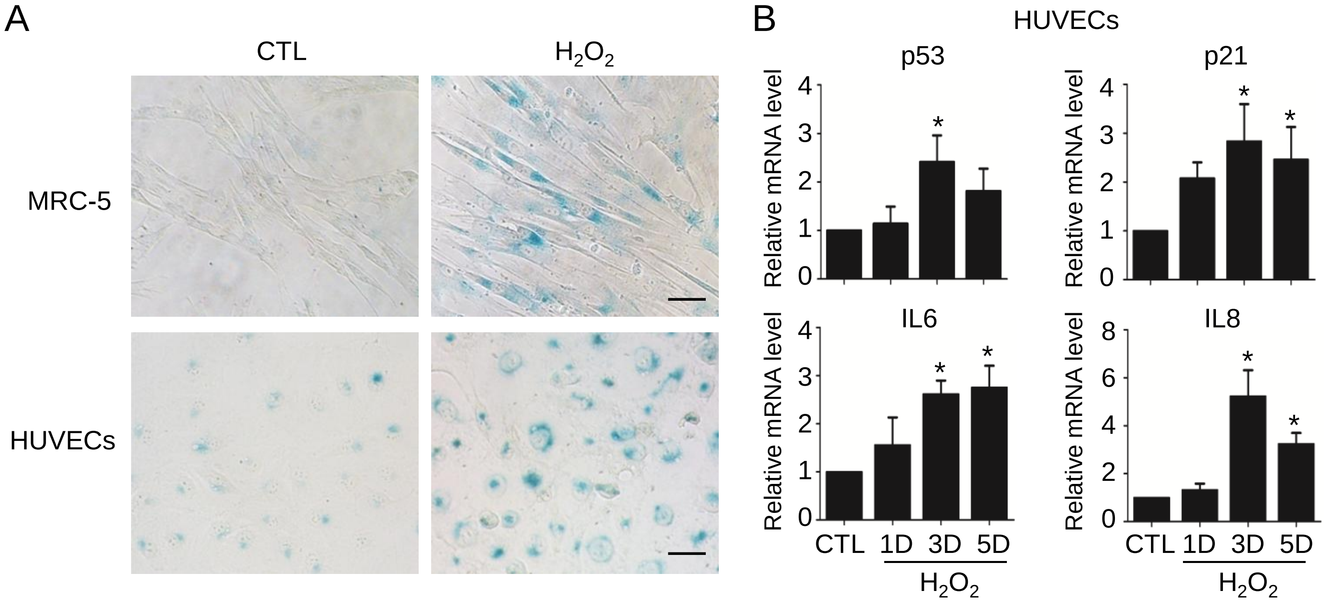

Figure S1. H_2O_2 induced senescence in MRC-5 cells and HUVECs. A and B: Cells were treated with H_2O_2 and incubated in complete medium without H_2O_2 for three days as described in the Experimental Procedures. (A) Representative images of SA-β-gal staining of MRC-5 and HUVECs. (B) Relative fold-changes in mRNA levels of senescence-associated genes, *p53*, *p21*, *IL6* and *IL8*, in HUVECs were monitored by qRT-PCR assays. * $p<0.05$ compared to the control (CTL). The bar represents 100 μm.

Figure S2

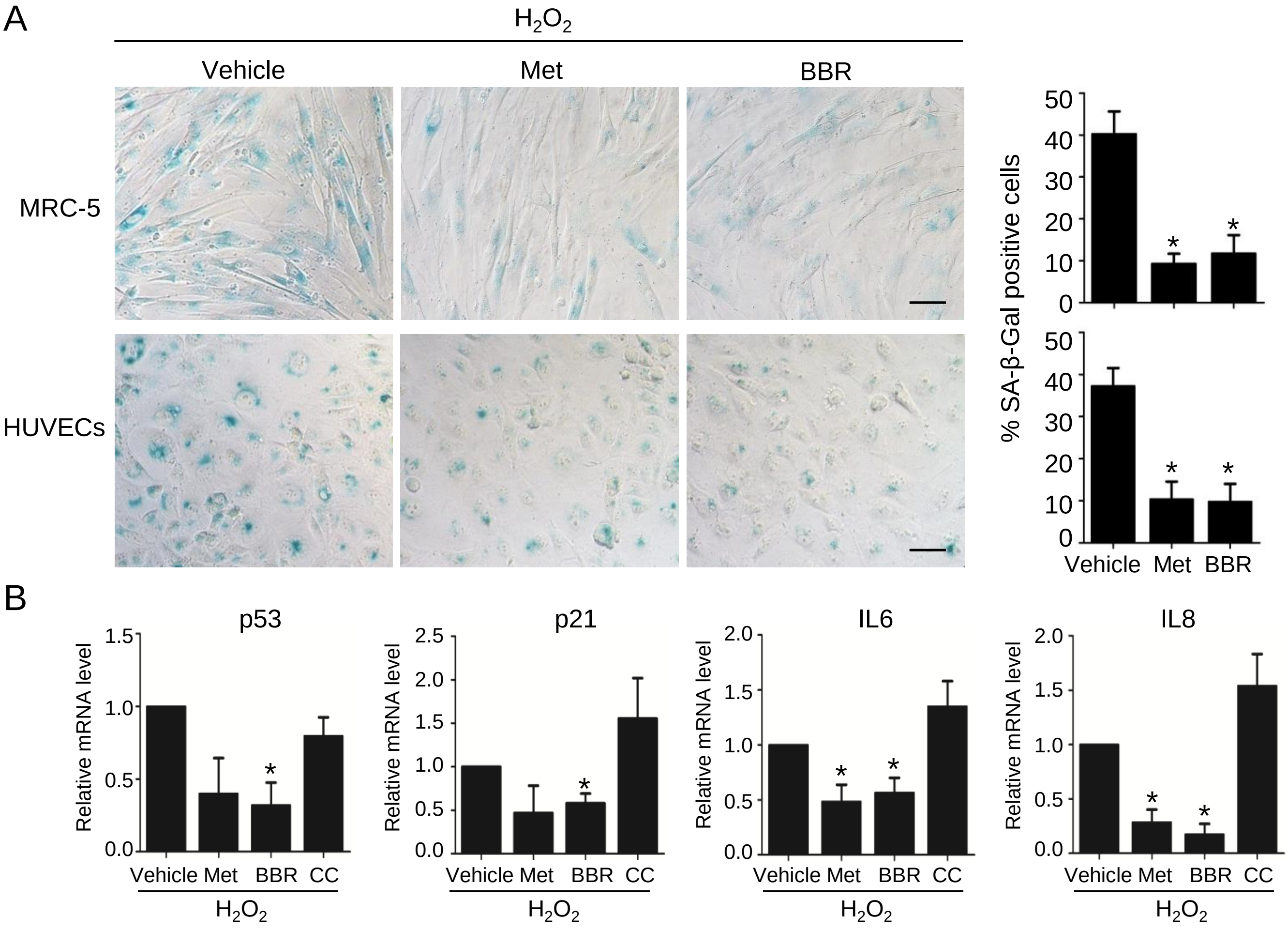

Figure S2. Activation of AMPK prevented H_2O_2-induced senescence in MRC-5 cells and HUVECs. H_2O_2-treated cells were incubated in complete medium with Met (10 mM) or BBR (10 µM) for 3 days. (A) Representative images of SA-β-Gal staining of the cells (left) and percentages of SA-β-gal-positive cells (right). The data from MRC-5 cells and HUVECs are shown in the upper and lower panels, respectively. (B) Relative fold-changes in mRNA levels of the genes encoding *p53*, *p21*, *IL6* and *IL8* in HUVECs were monitored by qRT-PCR. * $p< 0.05$ compared to the vehicle. The bar represents 100 µm.

Figure S3

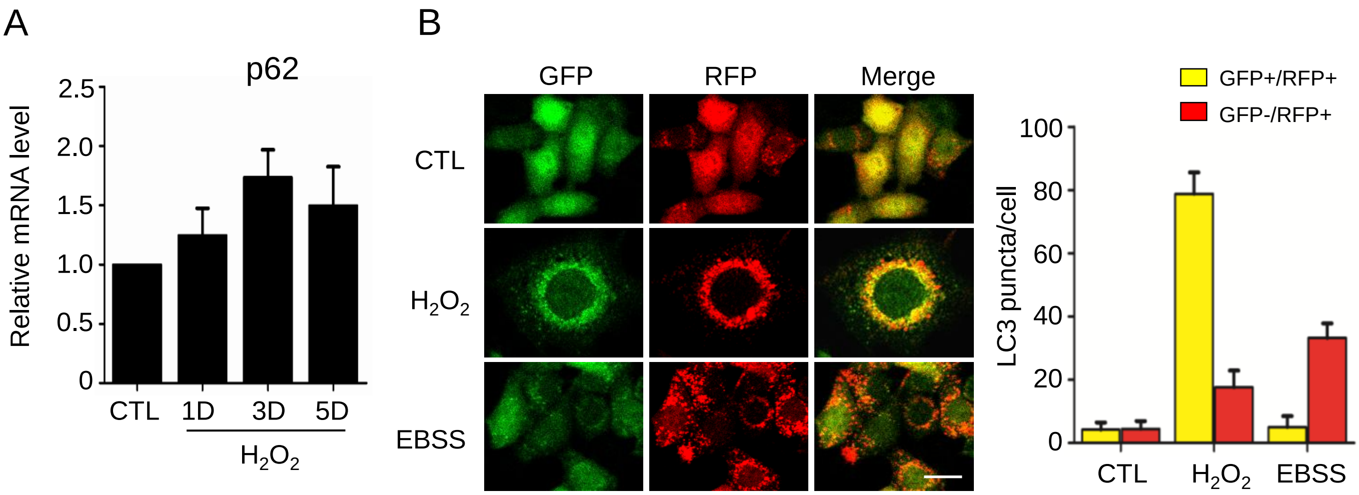

Figure S3. H_2O_2 induced decreased autophagic flux in NIH3T3 Cells. (A) NIH3T3 cells were treated with H_2O_2 and incubated in complete medium without H_2O_2 for 1-5 days. Relative fold-changes in the mRNA level of the *p62 /SQSTM1* gene were monitored by qRT-PCR. (B) Representative confocal fluorescent images of GFP-RFP-LC3-expressing cells, and the right panel shows the merged fluorescence (upper) and the percentages of cells with puncta-like LC3 (lower). The bar represents 20 μm.

Figure S4

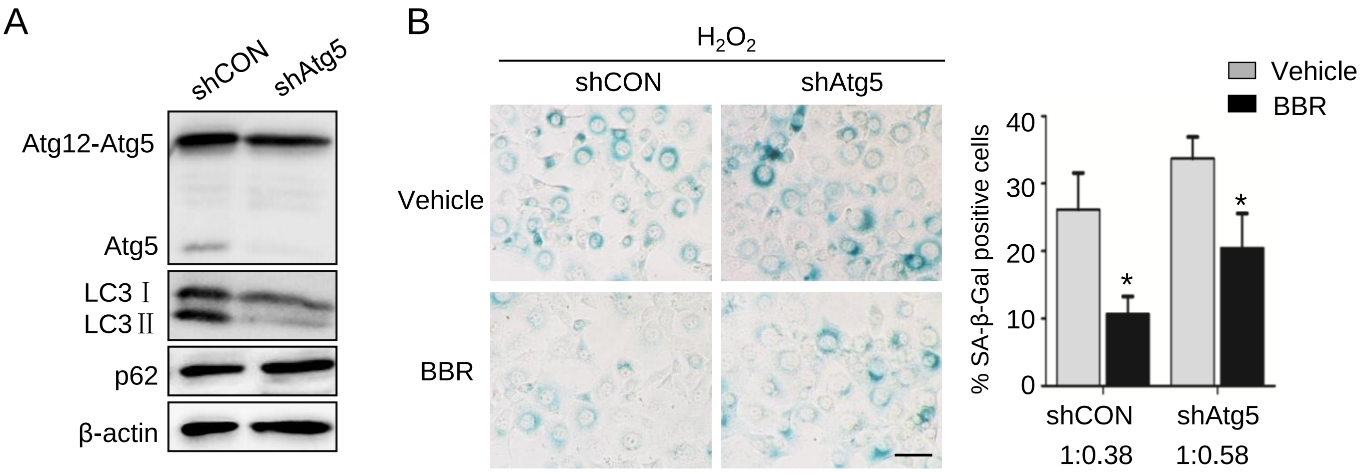

Figure S4. Atg5 knockdown attenuated the effects of BBR on protection against senescence. A to B: NIH3T3 cells infected with shAtg5 or a non-targeting shRNA (shCON). (A) Representative images from immunoblot assays against the Atg5, LC3 and p62 proteins. (B) Representative images of the SA-β-Gal staining of cells (left) and the percentages of SA-β-gal-positive cells (right). * $p < 0.05$ compared to the vehicle. The bar represents 100 μm.

Figure S5

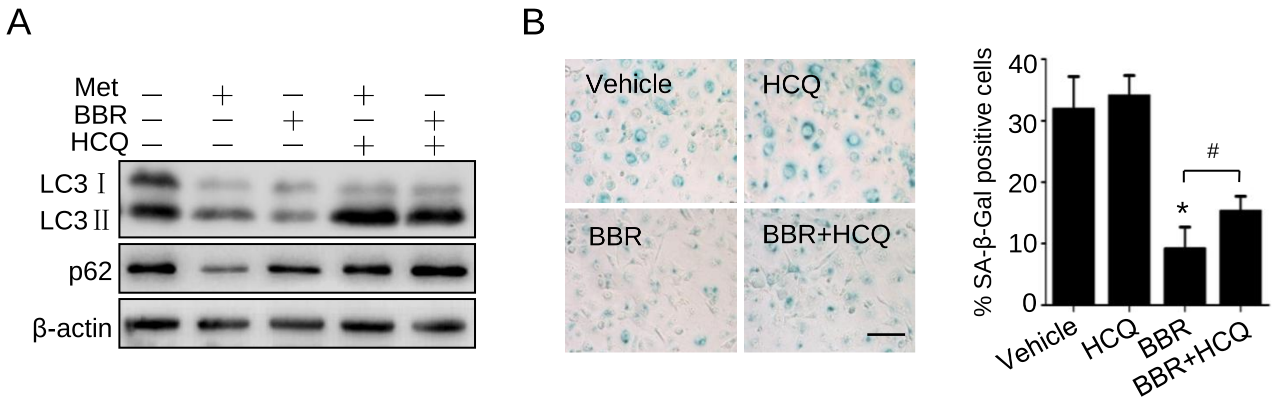

Figure S5. Activation of AMPK suppressed the impairment of H_2O_2-induced autophagic flux and decreased the senescence in HUVECs. A to C: H_2O_2-treated HUVECs were incubated with Met (10 mM), BBR (10 µM) and HCQ (2 µM) alone or in combination for 3 days. (A) Representative images from immunoblot assays against LC3 and p62 proteins. (B) Representative images of SA-β-Gal staining of the cells (left) and the percentages of SA-β-gal-positive cells (right). * $p< 0.05$ compared to the vehicle. # $p< 0.05$ compared to the indicated groups. The bar represents 100 µm.

Figure S6

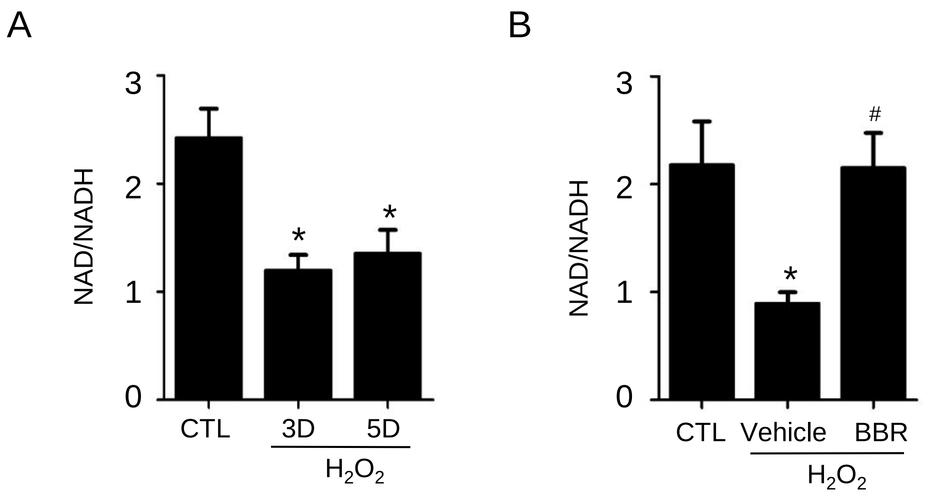

Figure S6. Activation of AMPK improved the redox status in senescent cells. (A) Cellular concentrations of NAD/NADH on day 3 or day 5 after H_2O_2 treatment in NIH3T3 cells incubated in normal medium. (B) H_2O_2-treated cells were incubated with BBR (10 µM) and CC (10 µM) for 3 days. The concentrations of NAD/NADH are shown. * $p<0.05$ compared to the CTL, # $p< 0.05$ compared to the vehicle.

Figure S7

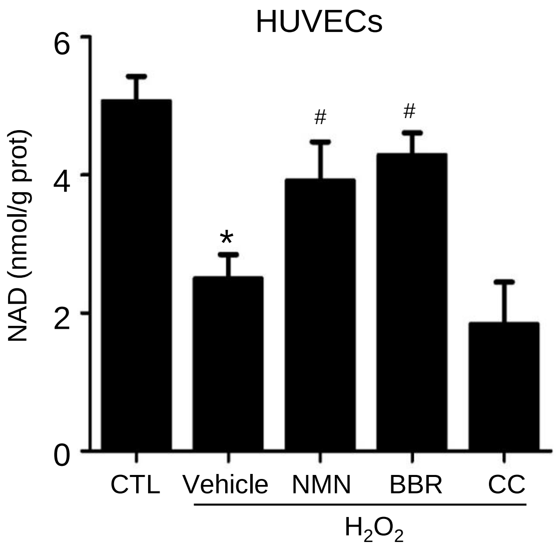

Figure S7. Activation of AMPK increased the NAD^+ level in HUVEC Cells. HUVECs were treated with H_2O_2 and incubated in complete medium with NMN (500 µM), BBR (10 µM) and CC (10 µM) for 3 days. The concentrations of NAD^+ are shown. * $p < 0.05$ compared to the control. # $p < 0.05$ compared to the vehicle.

Figure S8

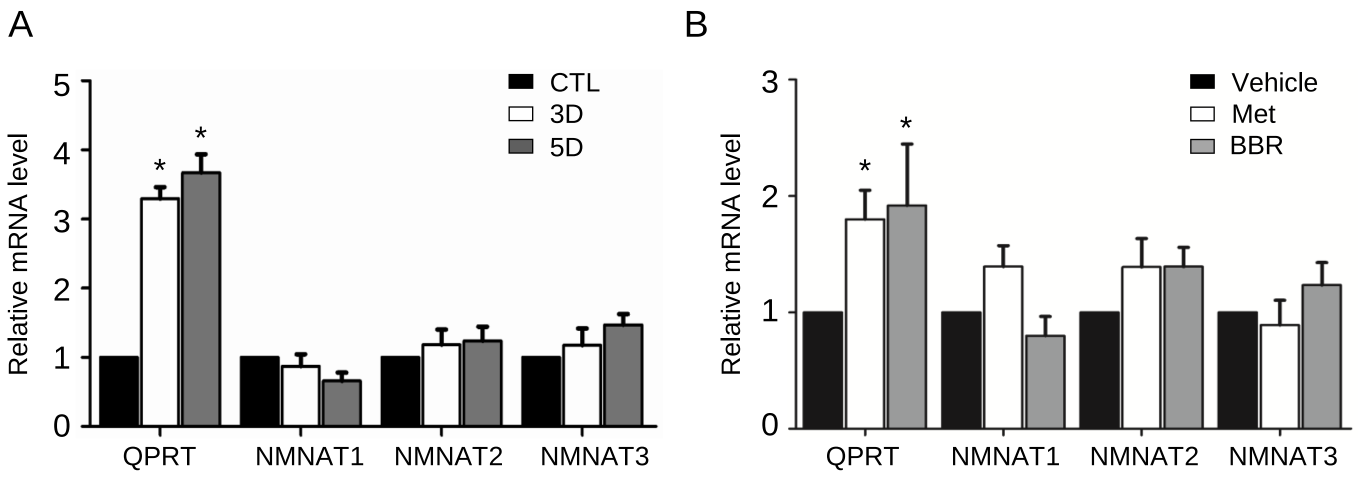

Figure S8. The mRNA level of QPRT is increased during senescence. (A) NIH3T3 cells were treated with H_2O_2 and incubated in complete medium without H_2O_2 for 3-5 days. The relative fold-changes in the mRNA level of *QPRT, NMNAT1, NMNAT2, NMNAT3* genes were monitored by qRT-PCR. (B) H_2O_2-treated cells were incubated with Met (10 mM), BBR (10 µM) for 3 days. The relative fold-changes in the mRNA level of *QPRT, NMNAT1, NMNAT2, NMNAT3* genes were monitored by qRT-PCR. * $p < 0.05$ compared to the control (A) or vehicle (B).

Figure S9

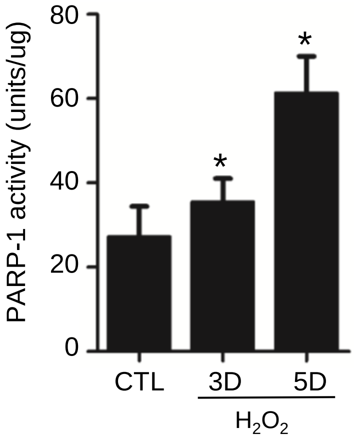

Figure. S9 The activity of PARP-1 is increased during senescence. NIH3T3 cells were treated with H_2O_2 and incubated in complete medium without H_2O_2 for 3-5 days. The activity of PARP-1 is shown. * $p< 0.05$ compared to the control.

Figure S10

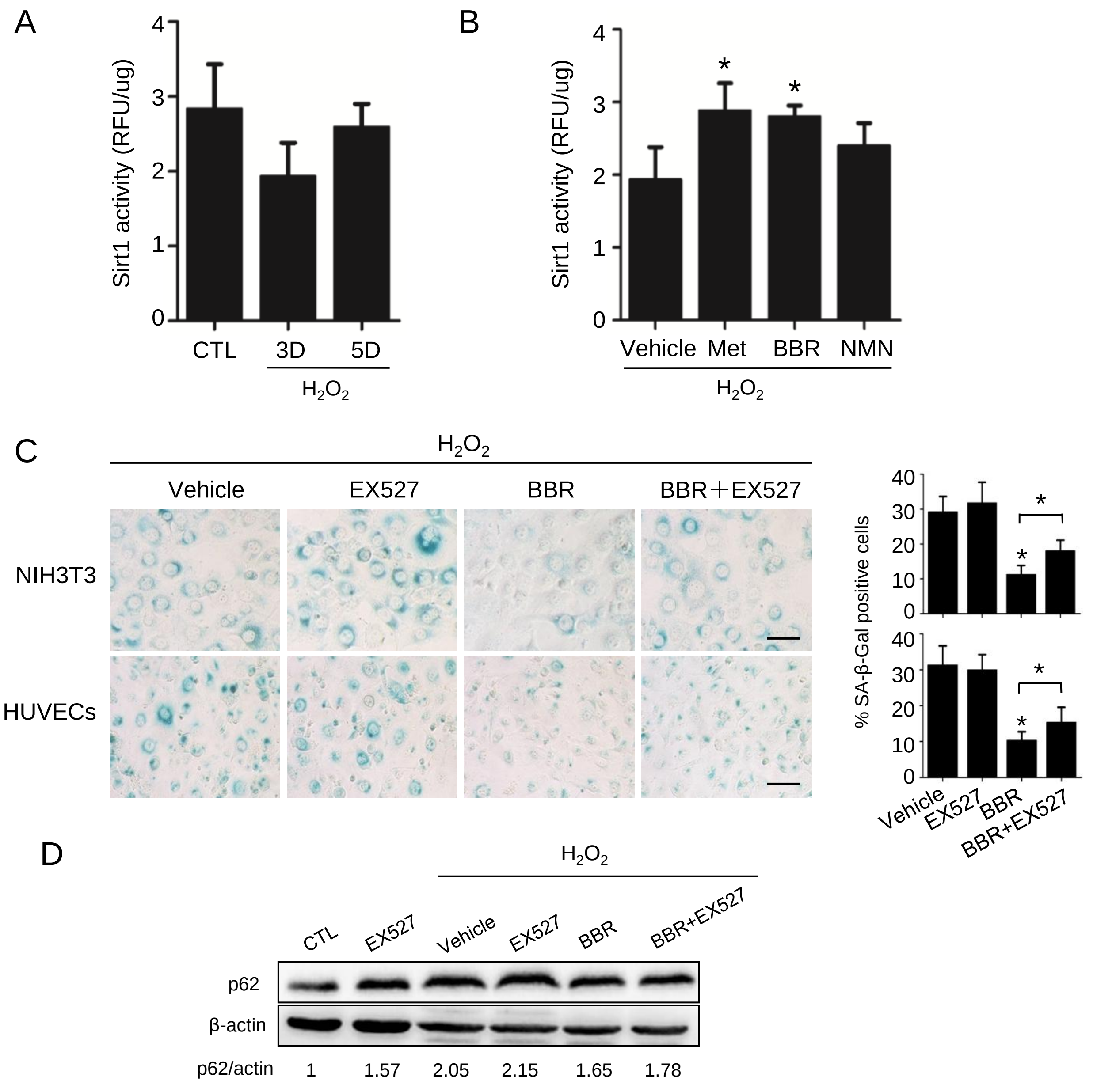

Figure S10. Sirt1 is involved in H_2O_2-induced senescence as a downstream mediator of AMPK and NAD^+ biosynthesis. (A) NIH3T3 cells were treated with H_2O_2 and incubated in complete medium without H_2O_2 for 3-5 days. The activity of Sirt1 is shown. (B) H_2O_2-treated NIH3T3 cells were incubated with Met (10 mM), BBR (10 μM) and NMN (500 μM) for 3 days. The activity of Sirt1 is shown. (C) H_2O_2-treated NIH3T3 cells and HUVECs were incubated with a Sirt1 inhibitor (EX527, 1 mM) and BBR (10 μM) alone or in combination as indicated for 3 days. Representative images of SA-β-Gal staining of cells (left) and the percentages of SA-β-gal-positive cells (right). (D) Representative images from immunoblot assays against p62 and β-actin. * $p<0.05$ compared to the vehicle (B) or indicated groups (C). The bar represents 100 μm.

Figure S11

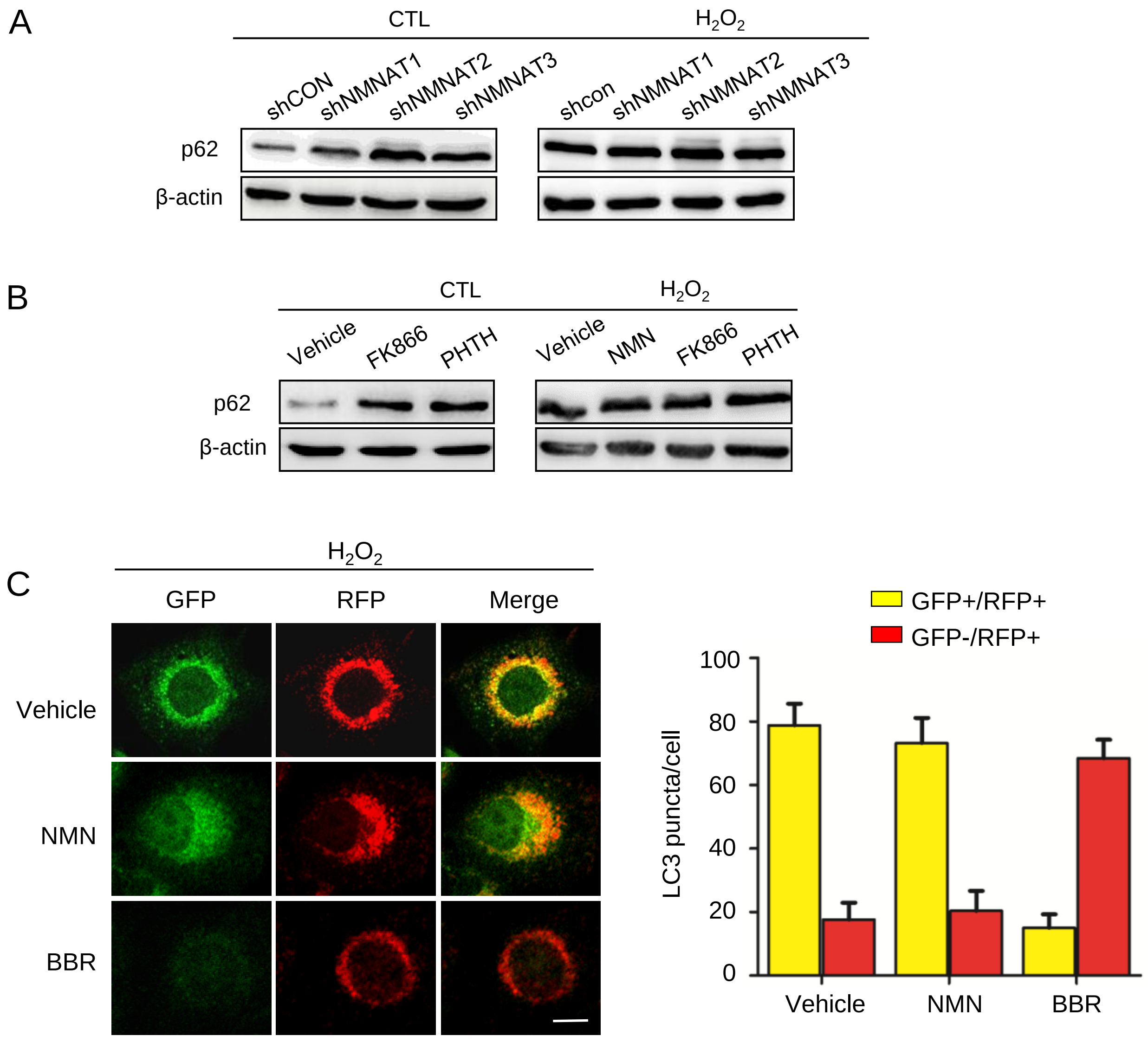

Figure S11. NAD+ homeostasis is required for maintaining the autophagic flux in normal cells, but not in senescent cells. (A) NIH3T3 cells infected with shRNAs were treated with or without H_2O_2 and cultured for 3 days. Representative images from immunoblot assays against p62 and β-actin are shown. (B) H_2O_2-treated or untreated NIH3T3 cells were incubated with FK866 (5 nM), PHTH (1 mM), and NMN (500 µM) for 3 days. Representative images from immunoblot assays against p62 and β-actin are shown. (C) Representative confocal fluorescent images of GFP-RFP-LC3-expressing cells. The right panel showed the merged fluorescence (left), and the percentages of cells with puncta-like LC3 expression were determined (right). The bar represents 20 µm.

Figure S12

Fig 1E

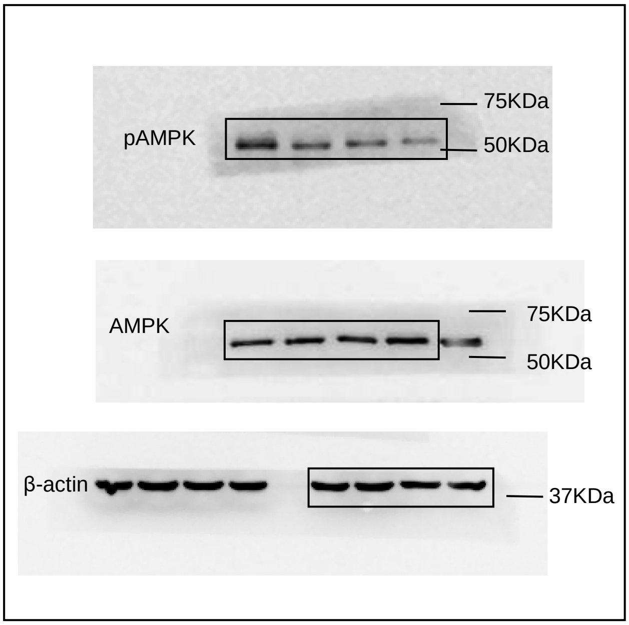

Fig 3B

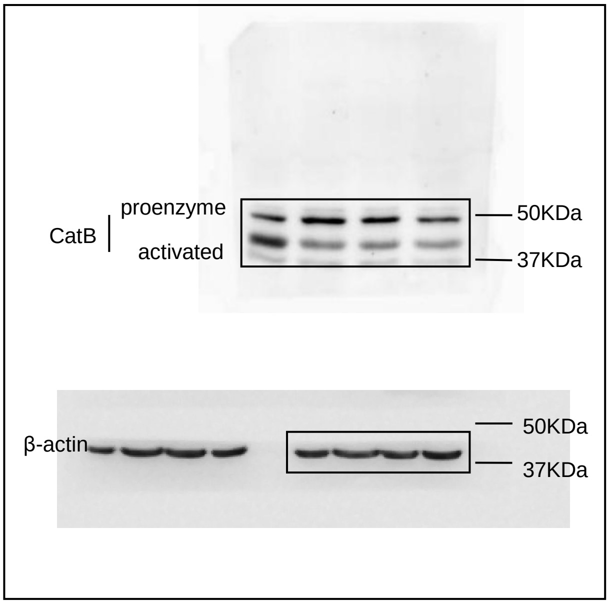

Fig 3H

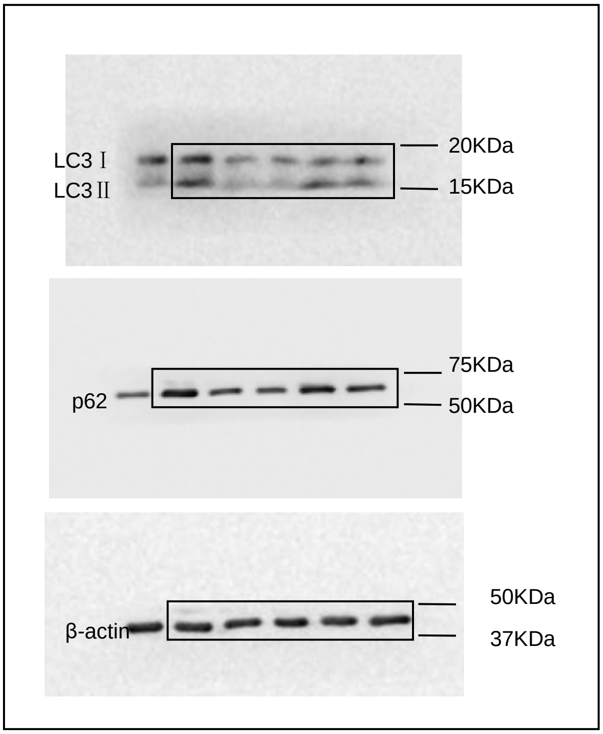

Figure S12. Unprocessed images of western-blot.
