# Supplementary material for: AMPK activation protects cells from oxidative stress‐induced senescence via autophagic flux restoration and intracellular NAD + elevation
Source: Aging Cell. 2016 Feb 18;15(3):416–27. doi: 10.1111/acel.12446 (PMC4854918; doi:10.1111/acel.12446)
Supplement: Supplementary file 2 — Table S1 Primers for real‐time qRT‐PCR. [file ACEL-15-416-s002.docx]

TableS1 Primers for real-time qRT-PCR

| Gene | Forward primer | Reverse primer |
| --- | --- | --- |
| *(m) p21* | 5’-GTGGCC TTGTCG CTG TCT T-3’ | 5’-GCGCTTGGAGTGATAGAAATCTG-3’ |
| *(m) IL6* | 5’-CTGCAAGAGACTTCCATCCAG-3’ | 5’-AGTGGTATAGACAGGTCTGTTGG-3’ |
| *(m) IL8* | 5'-CTCTTGGCAGCCTTCCTGATTT-3' | 5'-CGCAGTGTGGTCCACTCTCAAT-3' |
| *(m) CPT-1* | 5’-ATGGCAGAGGCT CACCAAGC-3’ | 5’-GATGAACTTCCAGGAGTGC-3’ |
| *(m) FAS* | 5’-GTAAGTTCTGTGGCTCCAGAG-3’ | 5’-GCCCTCCCGTACACTCACTC-3’ |
| *(m) STSTM1/p62* | 5’-AGGATGGGGACTTGGTTGC-3’ | 5’-TCACAGATCACATTGGGGTGC-3’ |
| *(m) GNS* | 5’- CGGTGTGCGGCTATCAGAC-3’ | 5’-CAGGGCATACCAGTAACTCCA-3’ |
| *(m) LAMP1* | 5’-CAGCACTCTTTGAGGTGAAAAAC-3’ | 5’-ACGATCTGAGAACCATTCGCA-3’ |
| *(m) QPRT* | 5’-CCGGGCCTCAATTTTGCATC-3’ | 5’-GGTGTTAAGAGCCACCCGTT-3’ |
| *(m) NAMPT* | 5’-CCTGGTATCCAATTACAGTGG C-3’ | 5’-CCAAATGAGCAGATGCCCCTAT-3’ |
| *(m) NMNAT1* | 5’-TGGCTCTTTTAACCCCATCAC-3’ | 5’-TCTTCTTGTACGCATCACCGT-3’ |
| *(m) NMNAT2* | 5’-TTGTAGATGAGAACGCCAACC-3’ | 5’-GTACCACGACGATCCCAAAGT-3’ |
| *(m) NMNAT3* | 5’-AGATGATGC CCT CAA TCA CCT-3’ | 5’-CCTGTGGTTCCTTCAACCCC-3’ |
| *(h) p53* | 5’-ACAAGGTTGATGTGACCTGGA -3’ | 5’-TGTAGACTCGTGAATTTCGCC -3’ |
| *(h) p21* | 5’-CGATGGAACTTCGACTTTGTCA-3’ | 5’-GCACAAGGGTACAAGACAGTG-3’ |
| *(h) IL6* | 5’-ACTCACCTCTTCAGAACGAATTG-3’ | 5’-CCATCTTTGGAAGGTTCAGGTTG-3’ |
| *(h) IL8* | 5’-TTTTGCCAAGGAGTGCTAAAGA-3’ | 5’-AACCCTCTGCACCCAGTTTTC-3’ |
| *18S* | 5’-TTGACGGAAGGGCACCACCAG-3’ | 5’-GCACCACCACCACGGAATCG-3’ |
