# Supplementary material for: AMPK activation protects cells from oxidative stress‐induced senescence via autophagic flux restoration and intracellular NAD + elevation
Source: Aging Cell. 2016 Feb 18;15(3):416–27. doi: 10.1111/acel.12446 (PMC4854918; doi:10.1111/acel.12446)
Supplement: Supplementary file 3 — Appendix S1 Extended Experimental Procedures. [file ACEL-15-416-s003.docx]

**Extended Experimental Procedures**

**DN-AMPKa1 expression cells generation**

DN-AMPKα1 expressing cells were generated by transfecting pDN-AMPKα1 plasmid (Jeong HW *et al.,* 2009) with Lipofectamine 2000 (Invitrogen, CA). Empty vector-transfected cells were generated in parallel.

**Acid** **phosphatase activity assay**

The total activity of acid phosphatase in NIH3T3 cells was quantified with a commercially available kit (Beyotime, Beijing, China) according to the manufacturer’s instructions. The average activity of acid phosphatase was normalized by lysosome mass that was measured via a process including Lyso-Tracker (Invitrogen, CA) staining of living cells, and then cells splitting with 1% Triton X-100 and fluorescence intensity reading by a fluorescence microplate reader. Experiments were repeated three times.

**Subcellular fractionation**

The cytoplasmic and nuclear proteins were extracted using a commercially available kit (Keygene, Nanjing, China) according to the manufacturer’s instructions. Briefly, at the end of the treatment, medium were discarded and cells were rinsed twice in PBS, harvested to a microcentrifuge tube in the presence of 1ml ice-cold PBS. Cells were collected by centrifugation at 300g for 5 min and PBS was dicarded. Cell lysis buffer (with 1 mM protease inhibitor, 1 mM DTT, 1 mM PMSF) with 1.5 time of packed cells volume was added to the cells , followed by vortexing for 15s and setting on ice for 30 min. Cytoplasic fraction was separated from nuclei by centrifugation at 16000g for 5 min at 4°C. The supernatant (cytoplasmic fraction) was transferred to a new tube, and the pellet (nuclear fraction) was resuspended in 100 µl of nuclei lysis buffer (with 1 mM protease inhibitor, 1 mM DTT, 1 mM PMSF) and vortexed for 15s, set on ice for 30min. Nuclear lysate was cleared by centrifugation at 16000g for 10 min at 4°C. Supernatant (nuclear protein exact) was transferred to a new tube. The protein concentration was measured by the BCA reagent (Cwbio, China), and samples were subsequently analyzed by immunoblotting.

**Immunofluorescence**

NIH3T3 cells were cultured on glass slides and fixed with cold 4% paraformaldehyde for10 minutes, and then permeabilized with 0.2% Triton X-100 in PBS for 10 min on ice. After 30 min of prehybridization in TBST-2% BSA and rinsing twice with PBS, the slides were incubated with primary antibody in 2% BSA for 1 h at room temperature, rinsed three times with PBS, and incubated with secondary antibodies produced in goat (diluted 1:200 in 2% BSA) for 1 h at room temperature in the dark. Nuclei were stained with DAPI. Images were taken under confocal fluorescence microscope.

**Lentiviral shRNA production**

Lentiviral shRNA constructs for Atg5, NMNAT1, NMNAT2 and NMNAT3 genes were purchased from GENECHEN (Shanghai, China). The target sequences were: AGAACCATACTATTTGCTT for Atg5; GTGGAAGTTGATACATGGGAA for NMNAT1; GCAGATATGGAGGTGATTGTT for NMNAT2; GAATGAGATCAGTGCCACATA for NMNAT3. Lenti-virus was produced by co-transfection of shRNA expression plasmids with plasmids encoding psPAX2 and pMD2.G using X-treme GENE HP (Roche) into 293T cells. Medium was changed 24 hr post-transfection and the medium containing virus was harvested after 72 hr, followed by a centrifugation at 10000g for 10 min. The supernatant was used to infect NIH3T3 cells in the presence of 5 µg/ml polybrene (Sigma-Aldrich, CA), or temperately stored at 4 °C for less than three days. Selection of resistant colonies was initiated 48 hr later using 20 µg/mL puromycin (Life Technology, CA).

**Poly (ADP-ribose) polymerase-1 (PARP-1) and Sirt1 activity assay**

To quantify PARP-1 and Sirt1 activity, commercially available PARP-1 assay kit (GENMED SCIENTIFICSINC) and Sirt1 PARP1 Enzyme Activity Assay Fluorometric kit (GENMED SCIENTIFICSINC) were used according to manufacturer’s instructions.

**Supplemental references**

Jeong HW, Hsu KC, Lee J-W, Ham M, Huh JY, Shin HJ, Kim WS, and Kim JB (2009) Berberine suppresses proinflammatory responses through AMPK activation in macrophages. *Am J Physiol Endocrinol Metab* 296, 955–964.
